# Mouse β-Defensin 3, A Defensin Inhibitor of Both Its Endogenous and Exogenous Potassium Channels

**DOI:** 10.3390/molecules23061489

**Published:** 2018-06-20

**Authors:** Yaoyun Zhang, Yonghui Zhao, Hongyue Liu, Weiwei Yu, Fan Yang, Wenhua Li, Zhijian Cao, Yingliang Wu

**Affiliations:** 1 State Key Laboratory of Virology, College of Life Sciences, Wuhan University, Wuhan 430072, China; zhangyaoyun@whu.edu.cn (Y.Z.); 2014202040050@whu.edu.cn (Y.Z.); 2017202040128@whu.edu.cn (H.L.); yu287@whu.edu.cn (W.Y.); y_fan@whu.edu.cn (F.Y.); whli@whu.edu.cn (W.L.); zjcao@whu.edu.cn (Z.C.); 2 Biodrug Research Center, Wuhan University, Wuhan 430072, China

**Keywords:** potassium channel, vertebrate defensin, mouse β-defensin 3, pharmacological feature, mouse Kv1.6 channel, inhibitor

## Abstract

The human defensins are recently discovered to inhibit potassium channels, which are classical targets of the animal toxins. Whether other vertebrate defensins are potassium channel inhibitors remains unknown. In this work, we reported that the mouse β-defensin 3 (mBD3) was a novel inhibitor of both endogenous and exogenous potassium channels. The structural analysis showed that mBD3 is the most identical to human Kv1.3 channel-sensitive human β-defensin 2 (hBD2). However, the pharmacological profiles indicated that the recombinant mBD3 (rmBD3) weakly inhibited the mouse and human Kv1.3 channels. Different from the pharmacological features of human β-defensins, mBD3 more selectively inhibited the mouse Kv1.6 and human KCNQ1/KCNE1 channels with IC_50_ values of 0.6 ± 0.4 μM and 1.2 ± 0.8 μM, respectively. The site directed mutagenesis experiments indicated that the extracellular pore region of mouse Kv1.6 channel was the interaction site of rmBD3. In addition, the minor effect on the channel conductance-voltage relationship curves implied that mBD3 might bind the extracellular transmembrane helices S1-S2 linker and/or S3-S4 linker of mouse Kv1.6 channel. Together, these findings not only revealed mBD3 as a novel inhibitor of both endogenous and exogenous potassium channels, but also provided a clue to investigate the role of mBD3-Kv1.6 channel interaction in the physiological and pathological field in the future.

## 1. Introduction

The defensins, which are widely produced by fungus, insects, invertebrates and vertebrate animals, are endogenous cationic peptides and effector molecules of the innate immune system because of their broad-spectrum antimicrobial activity [1,2,3]. Among the α-, β- and θ-defensin subfamilies, α- and β-defensins usually contain six or eight cysteine residues which intramolecularly form disulfide bonds [4]. Such structural features resemble the typical toxin peptides acting on potassium channels, which have induced researchers to confirm defensins as novel potassium channel inhibitors in recent years [5,6,7]. The defensins, such as plectasin, AtPDF2.3 and BmKDfin4 were successively identified as potassium channel blockers from fungi, plant and invertebrate scorpion, respectively [8,9,10]. In the vertebrate animals, the four human α- and β-defensins, HNP1, HD5, hBD1 and hBD2, were found to be Kv1.3 channel-selective inhibitors [11,12,13]. Different from HD5 and hBD1 as the blockers targeting the potassium channel pore region, HNP1 and hBD2 work as both modifiers and blockers binding the transmembrane helices S1-S2 linker and pore region of potassium channel, respectively [11,14]. This progress not only demonstrated the use of defensins as a new type of potassium channel inhibitor, but also suggested the functional complexity of defensins as modifiers and/or blockers of potassium channels, which indicates the essentiality of investigating more defensins from more species.

In this work, the interactions between the vertebrate mouse β-defensin and potassium channels were focused after human defensins in the world [11,12,13,14]. Using Kv1.3 channel-sensitive human defensin hBD2 as the molecular template, the sequence search and analysis indicated the mBD3 was the potential inhibitor of potassium channels. The pharmacological experiments showed that the recombinant mBD3 (rmBD3) could effectively inhibit endogenous Kv1.6 channel instead of mouse Kv1.1, Kv1.2, Kv1.3 channels. Besides mouse potassium channels, rmBD3 could also inhibit human KCNQ1/KCNE1 channel instead of other human potassium channels. Such novel pharmacological features not only demonstrated mBD3 was a novel inhibitor of both endogenous and exogenous potassium channels, but also revealed the functional diversity of vertebrate defensins, which would accelerate the more extensive research on defensin-potassium channel interactions in the future.

## 2. Method and Materials

### 2.1. Construction of rmBD3 Peptide Expression Vector

We obtained mBD3 nucleotide sequence at the National Center for Biotechnology Information (NCBI) gene bank (NCBI accession: NM_013756) and amplified the peptide fragment by overlapping PCR [6]. The PCR product was digested with *Kpn* I and *Xho* I and inserted into pET-32a (+) expression vector. The recombinant plasmid was transformed into bacteria *E. coli* Rosetta (DE3) cells for expression after confirmation by sequencing.

### 2.2. Expression and Purification of rmBD3 Fusion Protein

The prokaryotic expression system was used to express the rmBD3. After being transformed into *E. coli* Rosetta (DE3) cells, the bacteria cells were cultured at 37 °C in Luria-Bertani (LB) medium with ampicillin (100 μg/mL). 1 mM IPTG was added to induce peptide expression at the temperature of 25 °C when the bacteria reached its logarithmic growth phase. The bacteria cells were harvested after 8 to 10 h post-induction and resuspended into chilled 20 mM imidazole buffer containing 20 mM Tris-HCl, 0.5 M NaCl, 10% glycerinum (pH = 7.9). Then they were cracked using ultrasonic bath, the supernatant from the lysate was loaded to a nickel affinity column. The purified rmBD3 fusion protein was dialyzed with Enterokinase buffer containing 25 mM Tris-HCl, 50 mM NaCl, and 2 mM CaCl_2_ for 4 h and digested by Enterokinase (Sangon Biotech, China) at the temperature of 25 °C for at least 12 h but no more than 16 h. High performance liquid chromatograph (HPLC) on a C18 column (10 × 250 mm, 5 μm) (Elite-HPLC) was used to further purify and isolate the digested protein by using a linear gradient of 5% to 95% acetonitrile with 0.1% trifluoroacetic acid (TFA) in 60 min at a constant flow rate of 4 mL/min, and the absorbance was detected at 230 nm [15]. The molecular weight was confirmed by matrix-assisted laser desorption ionization time-of-flight mass spectrometry (MALDI-TOF-MS) [16]. The confirmed protein was sub-packed by pierce bicinchoninic acid (BCA) protein assay kit (Thermo Fisher Scientific, Pittsburgh, PA, USA) and stored at −80 °C refrigerator.

### 2.3. The Sources of Potassium Channels

The pRc/CMV-hKv1.3 vector was kindly provided by Prof. Stephan Grissmer (University of Ulm, Ulm, Germany) and Prof. Olaf Pongs (Zentrum für Molekulare Neurobiologie der University Hamburg, Hamburg, Germany). The cDNAs encoding human Kv1.1, Kv1.2, hERG and KCNQ1/KCNE1 channels were subcloned into the vector pIRES2-EGFP (TaKaRa Clontech, Mountain View, CA, USA) for coexpression with the green fluorescent protein (GFP), and the other channels were subcloned into vector pcDNA 3.1(+) (TaKaRa Clontech, Mountain View, CA, USA) for coexpression with GFP. The constructs were verified by DNA sequencing (Sangon Biotech, Shanghai, China). The mutants Asp400, Asp401, Val402, Asp403, Asp411, Met428 and Tyr429 on the pore region of wild type mouse Kv1.6 channel plasmids were mutated into Ala with QuikChange Site-Directed Mutagenesis Kit (Agilent Stratagene, Santa Clara, CA, USA). All the mutants were verified by DNA sequencing before being used (Sangon Biotech, China).

### 2.4. Cell Culture and Transfection

Human embryonic kidney 293 (HEK293) cells were cultured Dulbecco’s modified Eagle medium (Thermo Fisher Scientific, Pittsburgh, PA. USA) with 10% heat-inactivated fetal calf serum supplemented with penicillin (100 units/mL) and streptomycin (100 μg/mL) in a humidified 5% CO_2_ incubator at 37 °C. Plasmids were transfected into HEK293 cells using the TurboFect in vitro Transfection Reagent (Thermo Fisher Scientific, Pittsburgh, PA, USA). Potassium currents were recorded after transfection for 1 to 3 days and positive cells were selected based on the presence of GFP fluorescence.

### 2.5. Electrophysiological Recordings and Data Analysis

Electrophysiological experiments were carried out at room temperature using the whole-cell recording mode by EPC10 patch clamp amplifier (Heka Elektronik, Lambrecht, Germany), which was controlled by Patchmaster software version 2 × 65 (Heka Elektronik, Holliston, MA, USA) [5,6,13]. For all the channels, HEK293 cells were bathed with mammalian Ringer’s solution contained 5 mM KCl, 140 mM NaCl, 10 mM HEPES, 2 mM CaCl_2_, 1 mM MgCl_2_ and 10 mM D-glucose (pH adjusted to 7.4 with NaOH). When rmBD3 was applied, 0.01% BSA was added to the Ringer’s solution. A multichannel microperfusion system MPS-2 (INBIO Inc., Wuhan, China) was used to exchange the external recording bath solution. The internal pipette solution contains 140 mM KCl, 1 mM MgCl_2_, 1 mM EGTA, 1 mM Na_2_ATP and 5 mM HEPES (pH adjusted to 7.4 with NaOH). All channel currents were elicited by depolarizing voltage steps of 200 ms from the holding potential −80 mV to +50 mV. Currents were typically digitized at 20 kHz and filtered at 2.9 kHz (Bessel). Electrode resistances were 3.0–5.0 MΩ, and the series resistance was usually compensated by 85%. The currents through Kv channels [6] and KCNQ1/KCNE1 channel [17] were recorded according to previously published references.

The IC_50_ values were obtained by fitting a modified Hill equation to the data with the formula: *I*_rmBD3_/*I*_control_ = 1/(1 + [*rmBD3*]/IC_50_), where [*rmBD3*] is the concentration of rmBD3, *I_rmBD3_* and *I_control_* are the peak currents in the presence and absence of rmBD3 at four different concentrations. The conductance of mouse Kv1.6 channel was calculated by the formula: *G* = *I_peak_*/(*V* − *E*_Kv1.6_), where *I_peak_* is the peak current, *E_Kv1.6_* is the reversal potential of the Kv1.6 channel. The conductance-voltage (G-V) curves were generated by the measured peak currents and fitted with a V_50_-V equation using the formula: *G/G*_max_ = 1/(1 + *exp* [(*V* − *V*_50_)/*k*]), where *G* is the conductance of the channels, *G*_max_ is the maximal channel conductance, *V* is the membrane voltage, *V*_50_ is the voltage of half-maximal activation, and *k* is the slope factor. The data were presented as the means ± SE of at least three times repeated experiments. Sigmaplot 12.5 and GraphPad Prism 5 software were used for analysis.

## 3. Results

### 3.1. Structural Analysis of mBD3 as Potential Potassium Channel Inhibitor

**Figure 1:**
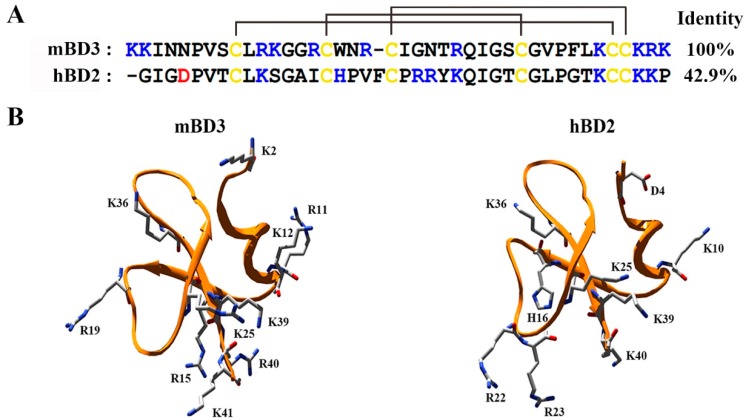
Sequence and structural analysis of mBD3 and the Kv1.3 channel-sensitive human defensin hBD2. (**A**). Amino acid residue sequence alignment analysis of mBD3 and hBD2. (**B**). The 3D structures of mBD3 and hBD2 (PDB code: 1FD4). The structure of mBD3 was predicted by the SWISS-MODEL server, and the positively and negatively charged residues were labeled.

So far, only two vertebrate β-defensins hBD1 and hBD2 were reported to work as Kv1.3 inhibitors with IC_50_ values of 11.8 μM and 22.0 pM, respectively [12,13]. Here, the Kv1.3 channel-sensitive hBD2 was used as the molecular template to search potential mouse β-defensin from the biological databases. As shown in Figure 1A, the mouse β-defensin mBD3 was obtained with 42.9% identity to hBD2. Besides the sequence similarity, the distribution of six cysteine amino acid residues was also identical. In order to better understand the structural feature of mBD3, its structure was modeled by the template of hBD2 (PDB code: 1FD4) (Figure 1B). Structural comparison of mBD3 and hBD2 showed the similar distribution of the positively charged amino acid residues in the spatial structures, that is, most of them distributed around the C-terminal of mBD3 and hBD2. Since the positively charged amino acid residues often play a critical role in mediating animal toxins to bind potassium channel pore region with many negatively charged amino acids [6,18,19], these similar sequence and residue distribution between mBD3 and hBD2 suggested that mBD3 was a novel inhibitor of potassium channels.

### 3.2. Expression, Purification and Identification of Recombinant mBD3

**Figure 2:**
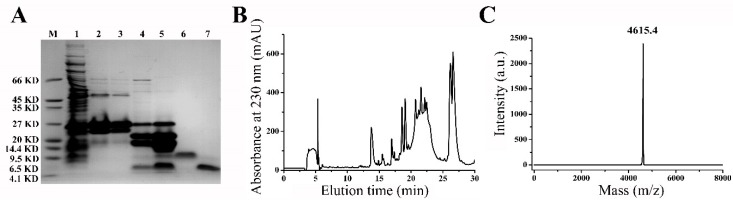
Expression, purification and identification of recombinant mBD3. (**A**) Fusion rmBD3 and purified rmBD3 were subjected to Tricine-SDS–PAGE. M, molecular weight markers; 1, the fusion rmBD3; 2, the purified fusion rmBD3 by nickel affinity column; 3, the purified fusion rmBD3 dialyzed with Enterokinase buffer; 4, Enterokinase digestion; 5, concentration; 6, the first peak of high performance liquid chromatograph after the 25 min; 7, the second peak of high performance liquid chromatograph after the 25 min. (**B**) Peak profile of rmBD3 purified by high performance liquid chromatograph. (**C**) The MALDI-TOF-MS result of rmBD3.

The prokaryotic expression method was used to express the recombinant mBD3 in this work. The target fragment was cloned into the expression vector pET-32a(+) to obtain a fusion protein containing an N-terminal His tag sequence. As shown in Figure 2A, the first nickel affinity purification showed that the fusion protein band was between 20 to 27 kDa, which was in line with the calculated molecular weight of HIS_6_-mBD3 with molecular weight of 21.7 kDa (Figure 2A, columns 1–3). Through the Enterokinase digestion for HIS_6_-mBD3 (Figure 2A, columns 4 and 5), the rmBD3 was obtained through the high performance liquid chromatograph by manual collection at 26–28 min, which is adjacent to the elution peak of another protein (Figure 2B). These two proteins were well validated by Tricine-SDS-PAGE analysis (Figure 2A, columns 6 and 7). Also, the MALDI-TOF-MS was further used to analyze rmBD3. It was found that the determined molecular weight was 4615.4 Da, which was corresponded to its calculated value of 4614.4 Da (Figure 2C). All these data showed that the recombinant mBD3 was successfully expressed.

### 3.3. Inhibition Effects of Potassium Channels by mBD3

**Figure 3:**
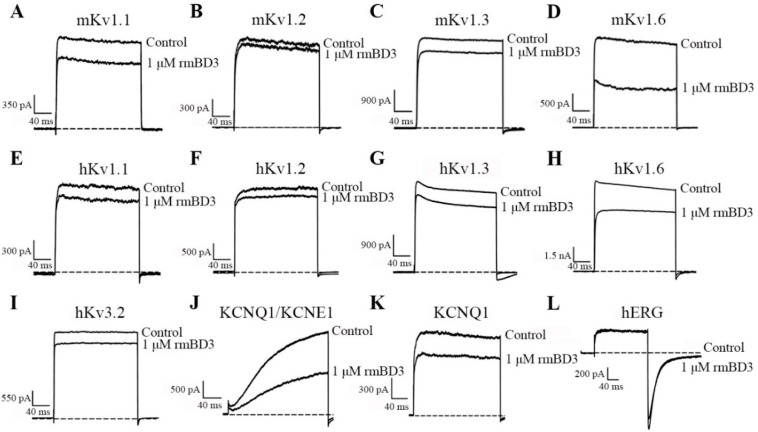
The rmBD3 inhibition of mouse and human potassium channels transfected in HEK293 cells. (**A**–**D**). The inhibitory effects of rmBD3 on mouse Kv1.1, Kv1.2, Kv1.3 and Kv1.6 channels, their current traces were shown in the absence (Control) or presence of 1 μM rmBD3, 1 μM rmBD3 inhibited potassium currents of 19.0 ± 2.2% for Kv1.1 (**A**), 4.9 ± 0.7% for Kv1.2 (**B**), 14.0 ± 3.7% for Kv1.3 (**C**) and 56.1 ± 4.2% for Kv1.6(**D**), respectively. (**E**–**L**). The inhibitory effects of rmBD3 on human Kv1.1, Kv1.2, Kv1.3, Kv1.6, Kv3.2, KCNQ1/KCNE1, KCNQ1 and hERG channels, their current traces were shown in the absence (Control) or presence of 1 μM rmBD3, 1 μM rmBD3 inhibited potassium currents of 8.8 ± 3.0% for Kv1.1 (**E**), 9.3 ± 0.8% for Kv1.2 (**F**), 14.6 ± 0.9% for Kv1.3 (**G**), 31.2 ± 3.6% for Kv1.6 (**H**), 11.6 ± 1.3% for Kv3.2 (**I**), 46.3 ± 4.2% for KCNQ1/KCNE1 (**J**), 26.1 ± 4.8% for KCNQ1 (**K**) and 14.7 ± 3.9% for hERG (**L**), respectively. Each channel was tested at least three times. Results were shown as the mean ± SE. All the curves were fitted with the Hill equation.

In order to verify whether the mBD3 could be an inhibitor of potassium channels, we examined it on different potassium channels subtypes through the patch clamp technique according to our previously procedure [5,6,13]. All the potassium channels were expressed in HEK293 cells, and the effects of rmBD3 on channels currents were measured. When the potential interactions between rmBD3 and mouse potassium channels were investigated, it was found that 1 μM rmBD3 could inhibit 19.0 ± 2.2%, 4.9 ± 0.6%, 14.0 ± 3.7% of mouse potassium currents mediated by Kv1.1, Kv1.2, Kv1.3, respectively (Figure 3A–C). These weak activities were different from previous human β-defensins [12,13], which prompted us to investigate whether other mouse potassium channels are possible targets of mBD3. It is interesting that we found 1 μM rmBD3 could inhibit 56.1 ± 4.2% of mouse Kv1.6 channel (Figure 3D), which indicated rmBD3 was a novel inhibitor of mouse potassium channels.

Besides the mouse potassium channels, we continued to investigate the potential binding of mBD3 towards human potassium channels. Similar to the insensitivity of mouse Kv1.1, Kv1.2 and Kv1.3 channels, the human Kv1.1, Kv1.2 and Kv1.3 channels also displayed weak sensitivity towards mBD3 (Figure 3E–G), which was distinct from human β-defensins [12,13]. Besides these three human potassium channels, human Kv1.6, Kv3.2, KCNQ1/KCNE1, KCNQ1 and hERG channels were also examined. As shown in Figure 3H–L, 1 μM rmBD3 showed weak activities on both Kv3.2 and hERG channels, but strong activities on Kv1.6 and KCNQ1/KCNE1 channels, whose 31.2 ± 3.6% and 46.3 ± 4.2% currents were inhibited by 1 μM rmBD3, respectively (Figure 3H–J,L). It was noticed that 1 μM rmBD3 could inhibit 26.1 ± 4.8% of KCNQ1 channel currents, which was much weaker than inhibition potency of rmBD3 on KCNQ1/KCNE1 channel (Figure 3J,K). Such functional differences suggested that auxiliary protein KCNE1 subunit potentially affected the rmBD3 binding. Together, all these pharmacological data indicated that mBD3 was a novel both endogenous and exogenous inhibitor of potassium channels.

### 3.4. mBD3 Dose-Dependently Inhibits Mouse Kv1.6 and Human KCNQ1/KCNE1 Channels

**Figure 4:**
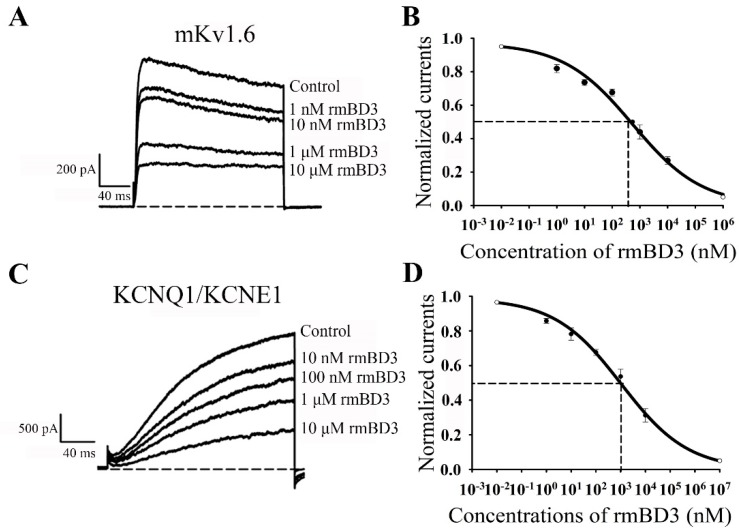
Concentration-dependent inhibition of mouse Kv1.6 channel and human KCNQ1/KCNE1 channel currents by rmBD3. (**A**) 18.1 ± 2.4%, 26.4 ± 1.8%, 56.1 ± 4.2% and 73.1 ± 2.2% of mouse Kv1.6 channel currents inhibited by 1 nM, 10 nM, 1 μM and 10 μM rmBD3, respectively. (**B**) Average normalized current inhibition by various concentrations of rmBD3 for mouse Kv1.6 channel. Hill equation fitting gives an IC_50_ value of 0.6 ± 0.4 μM. (**C**) 21.8 ± 3.6%, 32.4 ± 1.8%, 46.3 ± 4.2% and 68.8 ± 3.8% of human KCNQ1/KCNE1 channel currents inhibited by 10 nM, 100 nM, 1 μM and 10 μM rmBD3, respectively. (**D**) Average normalized current inhibition by various concentrations of rmBD3 for human KCNQ1/KCNE1 channel. Hill equation fitting gives an IC_50_ value of 1.2 ± 0.8 μM. The control currents amplitude in each experiment was fixed as 1 for the normalized currents and inhibition rates were compared; the R^2^ value were R^2^_mKv1.6_ = 0.98 and R^2^_KCNQ1/KCNE1_ = 0.96, respectively. Each channel was tested at least three times. Results are shown as the mean ± SE.

Then different concentrations of rmBD3 were used to examine the inhibition effects of mouse Kv1.6 and human KCNQ1/KCNE1 channel since it could more selectively inhibit these two potassium channels (Figure 3). As for mouse Kv1.6 channel, 1 nM, 10 nM, 1 μM and 10 μM rmBD3 were able to inhibit 18.1 ± 2.4%, 26.4 ± 1.8%, 56.1 ± 4.2% and 73.1 ± 2.2%, respectively (Figure 4A). Such concentration-dependent experiments showed that rmBD3 inhibited mouse Kv1.6 channel currents with an IC_50_ value of 0.6 ± 0.4 μM (Figure 4B). The pharmacological experiments also indicated that 10 nM, 100 nM, 1 μM and 10 μM were found to inhibit 21.8 ± 3.6%, 32.4 ± 1.8%, 46.3 ± 4.2% and 68.8 ± 3.8% of human KCNQ1/KCNE1 currents, respectively (Figure 4C), which showed that rmBD3 inhibited human KCNQ1/KCNE1 channel currents with an IC_50_ value of 1.2 ± 0.8 μM (Figure 4D). These data further indicated the inhibition potencies of mBD3 towards mouse Kv1.6 and human KCNQ1/KCNE1 channels.

### 3.5. The Pore Region of Mouse Kv1.6 Channel is Responsible for mBD3 Binding

**Figure 5:**
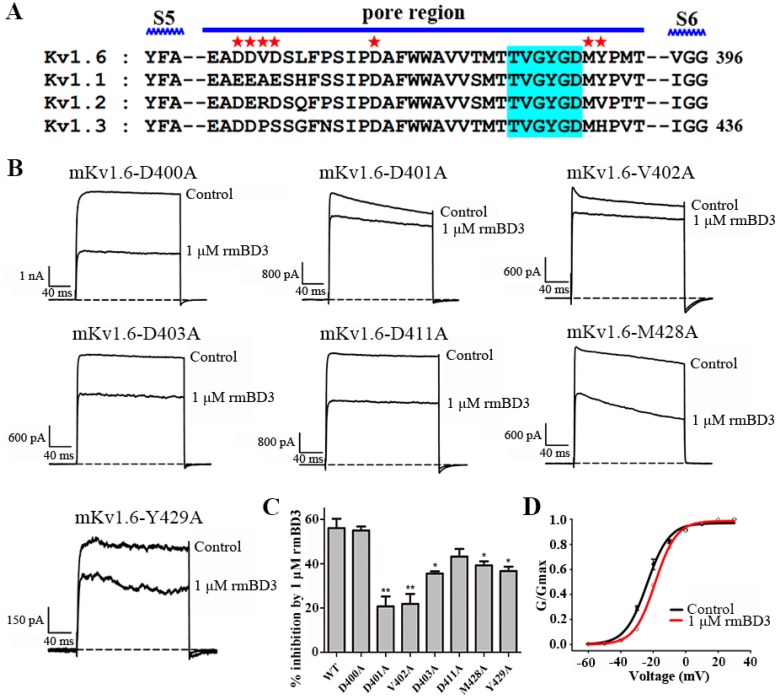
The mouse Kv1.6 channel pore region responsible for rmBD3 binding. (**A**) The amino acid sequences alignment for pore regions of mouse Kv1.6, Kv1.1, Kv1.2 and Kv1.3 channels, asterisks indicate amino acid residues of Kv1.6 channel that were changed in later functional studies. (**B**) Current traces of Kv1.6 channel mutants from the pore region in response to 1 μM rmBD3. (**C**) Averaged inhibition of wild-type and mutant Kv1.6 channel currents by 1 μM rmBD3, statistical significance was compared between the mutants and the wild-type Kv1.6 channel and was represented by asterisks marked correspondingly in the figures, where * *p* < 0.05, ** *p* < 0.01. (**D**) The effect of rmBD3 on activation G-V curves of Kv1.6 channel, solid lines indicate the Boltzmann fittings before (black) and after (red) treatment with 1 μM rmBD3, the V_50_ values were −23.8 ± 0.7 mV and −18.8 ± 0.5 mV, respectively; the *k* values were 6.8 ± 0.6 and 6.5 ± 0.4, respectively. The control currents amplitude in each experiment was fixed as 1 for the normalized currents and inhibition rates were compared. Each channel was tested at least three times. Results are shown as the mean ± SE.

The human β-defensins hBD1 and hBD2 displayed the distinct binding mechanisms as the potassium channel inhibitors: hBD1 only binding the pore region of human Kv1.3 channel, and hBD2 binding both the transmembrane helices S1-S2 linker and pore region of human Kv1.3 channel [12,13,14]. Here, we also wondered the binding interface of mouse Kv1.6 channel responsible for mBD3 inhibition. Firstly, the effect of mouse Kv1.6 channel pore region was investigated for rmBD3 binding. Based on the sequence alignment analysis among mouse Kv1.1, Kv1.2, Kv1.3 and Kv1.6 channels (Figure 5A), seven amino acid residues of mouse Kv1.6 channel, including Asp400, Asp401, Val402, Asp403, Asp411, Met428 and Tyr429 residues, were chosen for mutagenesis analysis. As shown in Figure 5B, the inhibition of mutant Kv1.6-D400A, Kv1.6-D401A, Kv1.6-V402A, Kv1.6-D403A, Kv1.6-D411A, Kv1.6-M428A and Kv1.6-Y429A channels by 1 μM rmBD3 were 54.9 ± 1.9%, 20.7 ± 4.5%, 21.8 ± 4.5%, 35.6 ± 1.0%, 43.2 ± 3.5%, 39.3 ± 1.8% and 36.7 ± 2.0%, respectively. These data indicated that Asp401 and Val402 residues in mouse Kv1.6 channel turret substantially lowered rmBD3 affinity, and other channel residues Asp403, Met428 and Tyr429 moderately decreased rmBD3 potencies (Figure 5B,C). These results proved that Kv1.6 channel pore region played an essential role in rmBD3 binding.

Secondly, the effect of rmBD3 on mouse Kv1.6 channel activation was also investigated through recognizing the transmembrane helices S1-S2 linker (such as human β-defensins hBD2 [14]) and/or S3-S4 linker (such as spider toxins [20]). Here, the channel conductance-voltage relationship (G-V) curves in the absence and presence of rmBD3 were measured to detect potential interaction between rmBD3 and mouse Kv1.6 channel. As shown in Figure 5D, the results showed that 1 μM rmBD3 did not significantly shift the G-V curve of Kv1.6 channel, and V_50_ values were −23.8 ± 0.7 mV and −18.8 ± 0.5 mV in the absence and presence of rmBD3, respectively. Such minor differences indicated that it was possible for rmBD3 to affect the activation of mouse Kv1.6 channel through targeting the extracellular transmembrane helices S1-S2 linker and/or S3-S4 linker. The differential effects of the extracellular domains in mouse Kv1.6 channel on rmBD3 binding demonstrated that pore region of mouse Kv1.6 channel is the key binding site of mBD3.

## 4. Discussion

The defensins are a large molecular resource, which are naturally produced by fungus, insects, invertebrates and vertebrate animals. So far, only several defensins were found to be the inhibitors of potassium channels in recent years, however, the functional roles of most defensins in binding potassium channels remains unknown, which further limits our understanding of defensins in physiological and pathological functions. For example, the binding of human defensins to human Kv1.3 channels in T cells could affect the cytokine secretion [11,13], which provides new evidence for defensin roles in the adaptive immunity. Therefore, the search of potassium channel-interacting defensins and its functional characterization undoubtedly remains an emerging area.

In this work, we reported the potassium channel-binding defensins from the second vertebrate animals, that is mouse β-defensin mBD3 since the discoveries of human defensins [11,12,13,14]. Although the mBD3 shared about 42.9% sequence identity with the Kv1.3 channel-selective human β-defensin hBD2 (Figure 1), the recombinant mBD3 showed very weak inhibition activity on both mouse and human Kv1.3 channels (Figure 3C,G). Among the mouse potassium channels investigated, the mBD3 could selectively and dose-dependently inhibit the currents of mouse Kv1.6 channels (Figure 3A–D and Figure 4A,B), and its IC_50_ value was 0.6 ± 0.4 μM. Such findings first demonstrated that mouse defensin was an endogenous inhibitor of own potassium channels. Besides the inhibition of mBDs towards endogenous Kv1.6 potassium channels, rmBD3 displayed weaker inhibition on human Kv1.6 channel, and some strong inhibition on human KCNQ1/KCNE1 channel with an IC_50_ value of 1.2 ± 0.8 μM (Figure 3E–L and Figure 4C,D). Such findings also indicated that mBD3 was an exogenous inhibitor of human potassium channels. These distinct pharmacological profiles of mouse mBD3 from human β-defensins undoubtedly expanded our knowledge of vertebrate defensing-potassium channel interactions.

The human β-defensins hBD1 and hBD2 presented differential binding mechanisms when they interacted with human Kv1.3 channels [12,13,14]. Here, the mouse mBD3 might adapt hBD2-like mechanism to recognize the pore region of mouse Kv1.6 channel, which was demonstrated by the site directed mutagenesis (Figure 5A–C). The gating effect of mBD3 was further supported by its minor effect on Kv1.6 channel activation (Figure 5D), which was similar to hBD2 modifier, different from hBD1 blocker [12,13]. During the scorpion toxin-potassium channel interactions, the electrostatic interactions usually play a critical role in their recognition process since the scorpion toxins as the basic peptides block the channel pore region with many acidic residues. As shown in Figure 1, the mBD3 is also a basic peptide with about 10 basic residues, which likely plays an important role in blocking the negatively-charged pore region of Kv1.6 channel through the dominant electrostatic interactions. However, the differential pharmacological profiles among hBD1, hBD2 and mBD3 were likely caused by the differential distribution of their potential functional residues (Figure 1), which deserves in-depth investigation in the future.

To our knowledge, the mouse Kv1.6 channel is mainly expressed in the cerebellum and brain [21,22], and mBD3 is widely distributed in various tissues and organs, including lung/trachea and bowel [23]. As a novel endogenous inhibitor of Kv1.6 channel, mBD3 would play a potential role in Kv1.6 channel-related physiological and pathological activities, which remains to be answered in the future. In addition, the human KCNQ1/KCNE1 channel, closely related to heart disease [24], was shortage of potent peptide blocker until now. Previously, we found that the scorpion venom could not effectively inhibit the KCNQ1/KCNE1 channel, and engineered MT2-2 peptide, an analog derived from a scorpion toxin BmKTX, was a weak blocker with IC_50_ value of 1.5 μM [25]. Here, we found that mBD3 could inhibit the human KCNQ1/KCNE1 channel with IC_50_ value of 1.2 ± 0.8 μM (Figure 4C,D). Two such peptides with these distinct structural features will help in discovering a potent blocker of the human KCNQ1/KCNE1 channel, as a potential therapeutic agent. Also, our work would stimulate the discoveries of more potassium channel-acting defensins from mouse and other vertebrate species, and enrich our understanding of defensin functions in the physiological and pathological activities.
